# Multimodal imaging of mild traumatic brain injury and persistent postconcussion syndrome

**DOI:** 10.1002/brb3.292

**Published:** 2014-12-02

**Authors:** Philip JA Dean, Joao R Sato, Gilson Vieira, Adam McNamara, Annette Sterr

**Affiliations:** 1 School of Psychology, University Of SurreyGuildford, UK; 2 Center of Mathematics, Computation and Cognition, Universidade Federal do ABCSão Paulo, Brazil; 3 NIF/LIM44, Departamento de Radiologia da Faculdade de Medicina da Universidade de São PauloSão Paulo, Brazil

**Keywords:** Cognitive tasks, mild traumatic brain injury, neuroimaging

## Abstract

**Background:**

Persistent postconcussion syndrome (PCS) occurs in around 5–10% of individuals after mild traumatic brain injury (mTBI), but research into the underlying biology of these ongoing symptoms is limited and inconsistent. One reason for this could be the heterogeneity inherent to mTBI, with individualized injury mechanisms and psychological factors. A multimodal imaging study may be able to characterize the injury better.

**Aim:**

To look at the relationship between functional (fMRI), structural (diffusion tensor imaging), and metabolic (magnetic resonance spectroscopy) data in the same participants in the long term (>1 year) after injury. It was hypothesized that only those mTBI participants with persistent PCS would show functional changes, and that these changes would be related to reduced structural integrity and altered metabolite concentrations.

**Methods:**

Functional changes associated with persistent PCS after mTBI (>1 year postinjury) were investigated in participants with and without PCS (both *n* = 8) and non-head injured participants (*n* = 9) during performance of working memory and attention/processing speed tasks. Correlation analyses were performed to look at the relationship between the functional data and structural and metabolic alterations in the same participants.

**Results:**

There were no behavioral differences between the groups, but participants with greater PCS symptoms exhibited greater activation in attention-related areas (anterior cingulate), along with reduced activation in temporal, default mode network, and working memory areas (left prefrontal) as cognitive load was increased from the easiest to the most difficult task. Functional changes in these areas correlated with reduced structural integrity in corpus callosum and anterior white matter, and reduced creatine concentration in right dorsolateral prefrontal cortex.

**Conclusion:**

These data suggest that the top-down attentional regulation and deactivation of task-irrelevant areas may be compensating for the reduction in working memory capacity and variation in white matter transmission caused by the structural and metabolic changes after injury. This may in turn be contributing to secondary PCS symptoms such as fatigue and headache. Further research is required using multimodal data to investigate the mechanisms of injury after mTBI, but also to aid individualized diagnosis and prognosis.

## Introduction

Traumatic brain injury (TBI) is a common cause of hospital admission in both the USA [1.6 million in 2003 (Rutland-Brown et al. 2006)] and the UK [156,000 in 2007 (Goodacre 2008)], with around 70–90% of those admissions having mild traumatic brain injury (mTBI) (Cassidy et al. 2004; Goodacre 2008). However, these figures are likely to underestimate the true incidence of mTBI as only a proportion of individuals who sustain an mTBI are admitted to hospital when visiting the emergency department [around 10–25% (Sosin et al. 1996; Bazarian et al. 2005)]. Instead they may be seen in private clinics, by primary care physicians, or may not seek or receive any medical attention (NCIPC 2003; Langlois et al. 2006). In the majority of individuals, the somatic, affective, and cognitive symptoms seen after mTBI (Ryan and Warden 2003) resolve within 3 months of injury (Korinthenberg et al. 2004; Lundin et al. 2006; Lannsjo et al. 2009; Sigurdardottir et al. 2009; Yang et al. 2009), but 5–10% of individuals go on to have persistent postconcussion syndrome [PCS (WHO 1992; Iverson 2005; Bigler 2008)] which can last a year or more postinjury (Killam et al. 2005; Sterr et al. 2006; Stulemeijer et al. 2007; Hessen et al. 2008; Dikmen et al. 2010). This is a potentially long-lasting problem as a large proportion of those reporting to hospital with mTBI are in younger age groups [e.g., around 30% are between 15 and 34 years old (Bazarian et al. 2005)].

The symptoms that make up PCS overlap with other clinical diagnoses such as depression (Iverson 2006), posttraumatic stress disorder (Bryant et al. 2009), and even occur to some extent in the general population (Wang et al. 2006; Fear et al. 2009; Dean et al. 2012). This has led some to believe that persistent PCS is psychogenic in origin (Mittenberg et al. 1992; Bailey et al. 2006; Mulhern and McMillan 2006; Belanger et al. 2010), especially as only a small proportion of those with mTBI present with lesions detectable by the standard neuroimaging techniques typically applied in hospital settings (Belanger et al. 2007; Lewine et al. 2007; Lee et al. 2008; Topal et al. 2008; Shenton et al. 2012). However, whilst there will be some psychological influence, it is becoming more apparent that there is also a subtle biological basis which can be observed in postmortem and advanced neuroimaging studies (Gonzalez and Walker 2011; Hunter et al. 2012; McDonald et al. 2012; Shenton et al. 2012; Dekosky et al. 2013; Smith et al. 2013).

One of these techniques, functional magnetic resonance imaging (fMRI), has been able to demonstrate functional differences in individuals after mTBI [(Witt et al. 2010; Mayer et al. 2011; Slobounov et al. 2011; Tang et al. 2011; Shumskaya et al. 2012), see (McDonald et al. 2012) for a review], and in relation to PCS symptoms (Smits et al. 2009; Pardini et al. 2010; Stevens et al. 2012). In some cases, functional alterations are observed even in the absence of differences in cognitive performance (McAllister et al. 1999, 2001; Chen et al. 2012) or ongoing symptoms (Johnson et al. 2012). fMRI is a useful tool for mTBI research as it can be employed to investigate the association between the cognitive symptoms (e.g., memory and attention deficits, slower processing speed) and the functional integrity of the neural areas underlying these behaviors after injury and during recovery. However, there have been relatively few fMRI studies with a specific focus on mTBI, and the majority of these look at the subacute or acute phase post-injury (McDonald et al. 2012). Most of the studies have used working memory [in particular n-Back (McAllister et al. 1999, 2001; Smits et al. 2009; Pardini et al. 2010; Chen et al. 2012)] and attention-related (Smits et al. 2009; Witt et al. 2010; Terry et al. 2012) tasks and found alterations in activation in the frontal lobe [particularly in left middle frontal gyrus (MFG) or dorsolateral prefrontal cortex (DLPFC)] as well as activation of more widespread task-unrelated areas (McAllister et al. 2001; Slobounov et al. 2010; Witt et al. 2010; Chen et al. 2012). Other studies have shown alterations in neural areas related to the default mode network (DMN) (Johnson et al. 2012; Mayer et al. 2012) and the medial temporal lobe (Stulemeijer et al. 2010), possibly related to impaired task-related deactivation. In addition, some studies have observed no group differences between mTBI and a control population (Elbin et al. 2012; Terry et al. 2012).

The inconsistent evidence base is probably caused by methodological factors, such as differences in the time since injury and task protocol, as well as the inherent heterogeneity of the mTBI population (Rosenbaum and Lipton 2012).

Each mild head injury is unique and will have different injury mechanisms and forces, as well as different preexisting psychological, demographic, and biological factors (Rosenbaum and Lipton 2012). This has led to some recent reviews of the area to ask for a more personalized injury prognosis, using multimodal imaging and cognitive testing to improve diagnosis and predict those at risk of poor outcome (Gonzalez and Walker 2011; Hunter et al. 2012; Irimia et al. 2012; McDonald et al. 2012; Shenton et al. 2012; Slobounov et al. 2012). Imaging techniques such as diffusion tensor imaging [DTI, (Shenton et al. 2012)] and magnetic resonance spectroscopy [MRS, (Lin et al. 2012)] have indicated subtle microstructural (e.g., diffuse axonal injury) and metabolic changes after mTBI. Acquiring fMRI, DTI, and MRS data for each individual would give a clearer indication of the severity of the injury. Biological changes after mTBI may only be visible in one of these imaging modalities for a specific individual, or there may be an interaction between multimodal data which allows detection of more subtle changes.

This study investigated the effects of mTBI in the long term (>1 year) after injury using fMRI, DTI, and MRS in a within-subject paradigm. The mTBI sample was split into those with ongoing PCS, and those without PCS in order to investigate the relationship between neuroimaging-derived biomarkers and symptom report. The tasks used during fMRI acquisition were n-Back to assess working memory [investigated in mTBI before (McAllister et al. 1999, 2001; Smits et al. 2009; Pardini et al. 2010; Chen et al. 2012)] and the paced visual serial addition task [PVSAT, used in TBI research with fMRI (Christodoulou et al. 2001; Maruishi et al. 2007)] to assess attention and processing speed. Both tasks contained four levels of difficulty which increased working memory load or speed of processing. The tasks were used in a previous cognitive study (Dean and Sterr 2013), which demonstrated cognitive deficits on both tasks only in those mTBI participants with ongoing PCS (>1 year post-injury). Previous work conducted by our group has demonstrated that individuals with PCS 1 year after mTBI show metabolic changes in right DLPFC (Dean et al. 2013) and reductions in structural integrity within the anterior corona radiata and splenium of the corpus callosum. This study aims to investigate functional differences 1 year post mTBI in the same sample as the previous neuroimaging studies (Dean et al. 2013), and their relationship with PCS symptoms, cognitive performance, and the previously observed metabolic and structural changes. It was hypothesized that only those individuals with mTBI and ongoing PCS would show activation differences, most likely in frontal lobe areas such as MFG and DLPFC. In addition, these changes would correlate with altered metabolite concentrations and reduced structural integrity.

## Materials and Methods

### Participants

Twenty five participants were included in this study, divided into three groups containing: 8 *mTBI + PCS* participants (suffered an mTBI and have persistent PCS); 8 *mTBI-PCS* participants (mTBI but no PCS); 9 *Control* participants (no history of brain injury and no PCS). One participant in the mTBI + PCS group had a corrupted n-Back behavioral file, so was not used in the fMRI analysis of this task. Group demographics and questionnaire data are shown in Table 1.

**Table 1:** Full participant information

Group	Age	Gender	RPQ	Cause of injury	Time since injury (years)
mTBI + PCS	19	M	31	Sport concussion	1.9
	19	M	15	Accidental fall	1.4
	21	F	23	Hit head upon object	1.0
	23	F	25	Motor vehicle accident	7.6
	24	F	35	Motor vehicle accident	2.2
	26	F	29	Accidental fall	2.9
	36	F	21	Hit head upon object	24.6
	37	F	23	Accidental fall	7.6
mTBI-PCS	22	M	12	Sport concussion	5.6
	23	F	18	Hit head upon object	7.8
	25	F	16	Hit head upon object	5.1
	26	F	16	Hit head upon object	1.2
	29	M	17	Accidental fall	7.8
	29	M	2	Accidental fall	3.1
	33	M	6	Sport concussion	4.3
	39	M	0	Hit head upon object	13.7
Control	18	F	3		
	18	M	7		
	19	M	6		
	20	F	5		
	20	M	3		
	22	M	3		
	23	F	8		
	25	F	8		
	32	F	0		

RPQ, Rivermead Postconcussion Questionnaire sum score.

Participants were recruited from a database generated by a convenience sampling of a large cross section of the general population during a previous study (Dean et al. 2012), which was also used in previous cognitive (Dean and Sterr 2013), MRS (Dean et al. 2013) and structural MRI studies. mTBI diagnosis was according to ICD-10 criteria [one or more of: dizziness/confusion; loss of consciousness <30 min; posttraumatic amnesia <24 h; (WHO 1992)] and PCS diagnosis was based on report that three (or more) of the symptom categories listed in the DSM-IV criteria (APA 1994) were more of a problem after injury. Postconcussion symptoms were measured using the Rivermead Postconcussion symptoms Questionnaire (RPQ) and Rivermead Postconcussion Questionnaire for Controls [RPQ-C; (Sterr et al. 2006)]. Control participants had no history of head injury, and low postconcussion symptom report, such that there could be no diagnosis of PCS had they had a history of head trauma. Inclusion criteria were that injury occurred at least 1 year prior to data collection (range in Table 1). Exclusion criteria were report of litigation, major invasive head injury, chronic pain, or other neurological conditions and visible lesions using standard structural MRI. The National Adult Reading Test [NART (Nelson 1982)] was taken as a measure of IQ. The study protocol was given a favorable opinion by the University of Surrey Ethics Committee, and informed consent was obtained.

### fMRI stimuli

**Figure 1:**
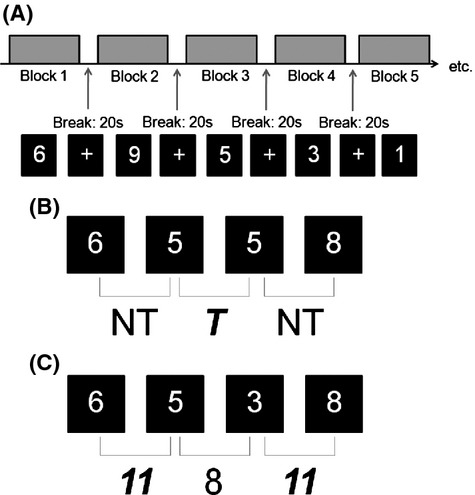
Illustration of (A) Study Design, (B) n-Back task and (C) PVSAT task. (A) illustrates the block design of the fMRI tasks (white numbers on black background) interspersed with rest screens (white cross on black background). Each block is preceded by an information screen telling the participant what type of block is coming next. At the end of the block, feedback (percent correct) is given, and the participant signals their wish to proceed to the next block by pressing any button. The example n-Back (B) is a 1-Back. First the number 6 appears, then 3 sec later the number 5, then 5 then 8. The task for 1-Back is to match the number currently presented to the number one previous. Five and 6 do not match, so the participant must respond using the nontarget button. However, 5 and 5 do match, so this requires a target button response. In the PVSAT example (C), the target number is 11 and the speed is 2s. First the number 6 appears, and then the number 5 appears 2 sec later, then 3, then 8. The task is to add the number on screen to the one previous: 5 + 6 = 11. This is the target number, so the participant must respond using the target button. Next, when the 3 appears, you must add it to the 5 (3 + 5 = 8). This is not the target number, so requires a nontarget button response.

Participants were presented with the same two behavioral tasks used in the prior cognitive study (Dean and Sterr 2013): the n-Back and the Paced Visual Serial Addition Task (PVSAT). Both the n-Back and PVSAT had four conditions or levels of difficulty (n-Back: 0-Back, 1-Back, 2-Back, 3-Back; PVSAT: 2.5 sec PVSAT, 2 sec PVSAT, 1.5 sec PVSAT, 1 sec PVSAT). The paradigm for both tasks was a block design (Fig.1A), with 12 blocks containing three randomly ordered repetitions of the four levels of difficulty. Task-related blocks started with an information screen detailing the difficulty level of the upcoming block presented for 1–3 sec (jittered) and were alternated with 20s rest blocks (white crosshair in center of black screen, see Fig.1A). A feedback screen was presented after each block detailing participant's performance. The order of presentation (n-Back/PVSAT) was counterbalanced across participants.

The stimuli for both tasks were sequentially presented white single digit numbers (between 1 and 9 inclusive, font: Arial, size 48) on a black background (see Fig.1). There were 30 of these stimuli per block, with 12 blocks in total. Within these 30 stimuli, there were 10 target stimuli presented randomly within each block, making 120 target stimuli and 240 nontarget stimuli for each task. Participants were asked to distinguish the target stimuli from the nontarget stimuli, and respond using their right index or middle fingers on an fMRI compatible button box. These buttons were counterbalanced as target and nontarget response buttons across the participants.

Although the tasks looked identical, there were differences in the interstimuli interval (n-Back: 3s; PVSAT: 2.5s, 2s, 1.5s, and 1s depending on condition) and participants had to perform different tasks to distinguish the target stimuli. In the n-Back task, participants were asked to press the target button when the number on screen matched the number observed one previous (1-Back), two previous (2-Back), or three previous (3-Back). For every other number that did not match, participants were asked to press the nontarget button (see Fig.1B). In the fourth condition (0-Back) a random number between 1 and 9 was designated as a target at the beginning of the block. Participants were requested to respond with the target button when they saw this number, and with the nontarget button otherwise.

For the PVSAT, participants were required to add the number on screen to the previously presented number. At the beginning of each block they were given a target number of 9, 10, or 11. If the addition equaled this target number, a target response was required. A nontarget response was required for every other addition (see Fig.1C). Each of the four conditions was presented with each of the three target numbers.

### MRI acquisition

Images were acquired using a 3T Siemens Trio MR Scanner (Siemens, Munich, Germany) in same sequence for each participant: T1-weighted structural, fMRI, DTI, MRS. Previous papers report the analysis of the MRS (Dean et al. 2013) data in detail. This paper looks at the relationship between these modalities, and with the fMRI data, in the same participants.

High-resolution 3D brain MRI images were obtained using a T1-weighted Magnetization Prepared Rapid Acquisition Gradient Echo (MPRAGE) pulse sequence (TR = 1830 ms; TE = 4.43 ms; Inversion Time = 1100 ms; flip angle = 11°; FOV = 256 mm; 176 slices; voxel size = 1 × 1 × 1 mm^3^; in-plane matrix = 256 × 256).

fMRI images were acquired using a Blood Oxygen Level Dependent (BOLD)-sensitive EPI sequence (TR = 2000 ms, TE = 30 ms, 90° flip angle). Thirty-five axial slices (FOV = 192 × 192 mm, 64 × 64 matrix, 3 mm thickness (33% gap), ascending interleaved slice acquisition).

Diffusion-Weighted Images (DWI) were acquired using a single-shot diffusion-weighted echo-planar imaging sequence, with diffusion gradients applied along 12 directions (b0 = 0 [1 image], b1 = 1000 smm^−2^ [12 images]; TR = 8900 ms, TE = 100 ms, number of averages = 4, 55 slices, voxel size = 2.5 × 2.5 × 2.5 mm^3^, in-plane matrix = 88 × 128, bandwidth = 2056 Hz, FOV = 320 × 220).

Single voxel MRS was performed over the right dorsolateral prefrontal cortex (DLPFC), using a PRESS sequence at short echo time [1.5 × 1.5 × 1.5 cm; echo time (TE) = 30 ms; repetition time (TR) = 1500 ms; CP coil; bandwidth = 2000 Hz; 2048 data points]. Both water-suppressed spectra (256 averages) and spectra without water suppression (16 averages) were acquired. The voxel was placed using T1-weighted axial and coronal structural scans and anatomical landmarks, in the area of DLPFC as reported in an fMRI n-Back meta-analysis (Owen et al. 2005). DLPFC was selected as the region of interest as it is differentially affected by mTBI, with differences observed in a number of fMRI studies. MRS was performed after a sustained activation of this area during the n-Back and PVSAT tasks. When placing the voxel, care was taken so that the voxel contained no cerebrospinal fluid, and extravoxel lipid saturation bands were used.

### Demographic and behavioral data analysis

For the participant information, a series of one-way ANOVAs, with post-hoc Bonferroni-corrected comparisons, were performed. Differences in gender between groups were investigated using a chi-square test. Behavioral data were analyzed using a mixed-model ANOVA with factor *difficulty level* (for n-Back: 3-Back, 2-Back, 1-Back, 0-Back; for PVSAT: 1s, 1.5s, 2s, 2.5s) and between-subjects factor of *group* (Control, mTBI-PCS, mTBI + PCS).

### fMRI data analysis

fMRI data analysis for each task was performed in the same way, using FEAT (fMRI Expert Analysis Tool), part of FSL [FMRIB, Oxford, http://fsl.fmrib.ox.ac.uk/fsl/fslwiki/ (Smith et al. 2004; Woolrich et al. 2009; Jenkinson et al. 2012)]. Preprocessing consisted of motion correction, removal of nonbrain structures, high-pass temporal filtering at 355s, spatial smoothing using a 5 mm FHWM Gaussian filter and mean-based intensity normalization. First-level time-series analysis was performed using FILM (FMRIB's Improved Linear Model) full mode setup, with gamma convolution and each task condition added as an explanatory variable (EV). The fMRI images were registered to the MNI standard brain via their individual T1-weighted structural images using FLIRT (FMRIB's Linear Image Registration Tool) and 12 degrees of freedom for the affine registration. Contrasts at the first level looked at the BOLD response difference between each task condition (n-Back: 0-, 1-, 2-, 3-Back; PVSAT: 2.5s, 2s, 1.5s, 1s) and rest, the difference between the least taxing condition in each task and the other more taxing conditions (n-Back: 0 vs. 1-Back, 0 vs. 2-Back, 0 vs. 3-Back; PVSAT: 2.5s vs. 2s, 2.5s vs. 1.5s, 2.5s vs. 1s) and the linear increase from the least taxing to the most taxing task ([−1.5 −0.5 0.5 1.5] for 0-, 1-, 2-, 3-Back and 2.5s, 2s, 1.5s, 1s PVSAT). Second-level analysis of group differences (Control vs. mTBI-PCS, Control vs. mTBI + PCS, mTBI-PCS vs. mTBI + PCS) in these first-level contrasts was performed using FLAME (FMRIB's Local Analysis of Mixed Effects; FLAME1 [Standard]). The *z*-statistic images were corrected for multiple comparisons using cluster thresholding (voxelwise *z*-statistic threshold: Z > 1.9; cluster probability threshold: *P* < 0.05).

For the sake of conciseness, the second-level group difference analysis reported in this manuscript focused on the contrast between the least and most taxing conditions (n-Back: 3 > 0-Back; PVSAT: 1 > 2.5s) in order to investigate the differences in the capacity of each group to respond to increased cognitive load. The main effect of task condition and contrasts between the less taxing conditions either yielded similar results or no significant group differences. However, the main effect of the least taxing condition (0-Back, 2.5s PVSAT) and the parametric (linear) contrast are presented in the results for each group separately. These serve as a reference of the typical BOLD response and parametric increase with task difficulty with which to interpret the group difference results.

Finally, the effect of postconcussion symptom report (indexed by RPQ score) on BOLD response (contrasts: n-Back: 3 > 0-Back; PVSAT: 1 > 2.5s) was investigated using a FLAME analysis across all groups (*n* = 25).

### Correlational analysis between fMRI, DTI, and MRS

Structural and metabolic MRI data were acquired at the same time as the functional data in the same group of participants. This enabled the investigation of the relationship between functional, structural, and metabolic data in the same injured brain, allowing a more comprehensive representation of the changes seen after mTBI, and their relationship with persistent PCS.

Previous publications detail the analysis results of the MRS (Dean et al. 2013) and DTI (P. J. A. Dean, J. R. Sato, G. Vieira, A. McNamara, A. Sterr, In Submission) data. However, in order to aid interpretation of the results presented in this paper, the analysis will also be detailed here. DTI analysis was performed with the Diffusion II toolbox (http://sourceforge.net/projects/spmtools) in SPM8. Diffusion-Weighted Images (DWI) were motion corrected, realigned to the mean image, normalized to MNI space, the gradient information, and the diffusion tensor eigenvalues and eigenvectors were calculated to generate the fractional anisotropy (FA) for each participant. FA maps were masked by “brainmask.nii” within SPM, and smoothed with a Gaussian kernel of FHWM = 8 mm. Second-level group analysis was carried out using two-sample *t*-tests within SPM8 to look at the effect of mTBI (mTBI [mTBI + PCS/mTBI-PCS] vs. Control), controlled for age at scan. In addition, the association between PCS and FA was investigated across participants using a one sample t-test with RPQ and age as covariates. The resulting statistical maps were thresholded at *P* < 0.001, uncorrected.

MRS spectra were processed using LC Model (Provencher 1993), using the default settings of water scaling, to obtain metabolite concentrations (total Creatine [Cr], total Choline [Cho], total NAA, all Cramér-Rao lower bound <15%), relative to water. Metabolite ratios within the DLPFC were also calculated: Cr/Cho.

Key indices were extracted from the functional, structural, and metabolic data of each participant, and represented regions (fMRI, DTI) or metabolites (MRS) which significantly differed between groups, or were significantly associated with PCS (indexed by RPQ Score). The associations between was then explored using a series of Pearson's partial correlations (age held constant) across participants (*n* = 25) and within mTBI participants (*n* = 16).

**Figure 2:**
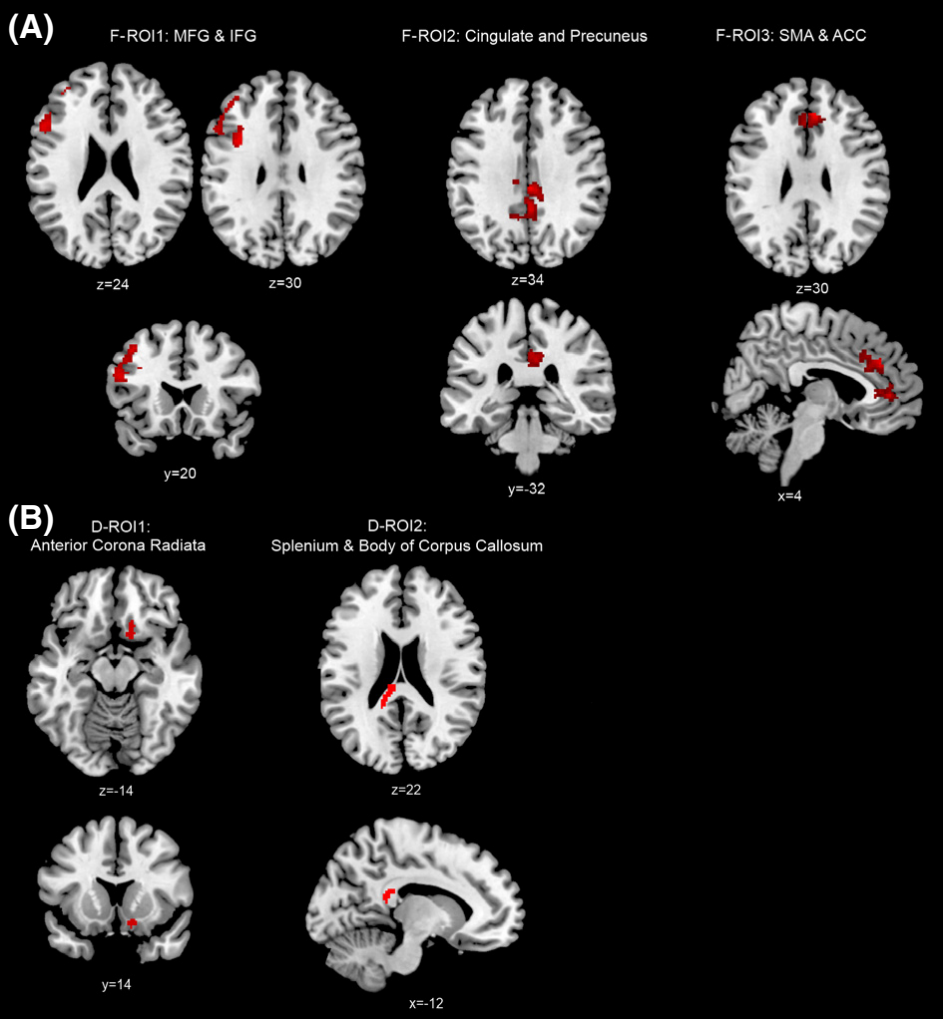
Regions of Interest for fMRI (F-ROI, A) and DTI (D-ROI, B).

The fMRI indices consisted of the *z*-statistic for each individual extracted from three fMRI regions of interest (F-ROI's, see Table 2). These F-ROIs were areas with significant BOLD response difference between groups, or significant association with PCS symptom report, and were chosen to represent changes in prefrontal areas (F-ROI1: Medial and Inferior Frontal Gyrus [MFG/IFG], 1 > 2.5s PVSAT), default mode network areas (F-ROI2: Posterior Cingulate Cortex [PCC] and Precuneus, 1 > 2.5s PVSAT correlation with RPQ) and attention-related areas (F-ROI3: Supplementary Motor Area [SMA] and Anterior Cingulate Cortex [ACC], 3 > 0-Back correlation with RPQ). ROI masks were created from the overlap between the *z*-statistic image for the contrast (Z > 1.9, group level analysis) and an AAL labeled mask of the main area of the cluster (Fig.2A)

**Table 2:** Correlation analysis: Regions of Interest

Modality	ROI	Area	Group	Correlation	Task	Condition Contrast
fMRI	F-ROI 1	Left MFG/IFG	Control > mTBI + PCS	–	PVSAT	1 > 2.5s
	F-ROI 2	PCC/Precuneus	–	RPQ (*n* = 25)	PVSAT	1 > 2.5s
	F-ROI 3	SMA/ACC	–	RPQ (*n* = 24)	n-Back	3 > 0-Back
DTI	D-ROI 1	Right ACR	Control >mTBI (*n* = 16)	–	–	–
	D-ROI 2	Splenium & Body of CC	–	RPQ (*n* = 16)	–	–

ROI, region of interest; F-ROI, fMRI ROI; D-ROI, DTI ROI; MFG, medial frontal gyrus; IFG, inferior frontal gyrus; PCC, posterior cingulate cortex; SMA, supplementary motor area; ACC, anterior cingulate cortex; ACR, anterior corona radiata; CC, corpus callosum; RPQ, Rivermead Postconcussion Questionnaire.

fMRI and DTI regions of interest used in Pearson's partial correlation analysis, for full map see Figure2. Correlation ROI from data across participants (*n* = 25 or *n* = 24) or only within mTBI participants (*n* = 16).

The DTI indices consisted of the average fractional anisotropy (FA) for each individual extracted from two DTI ROI's (D-ROI, see Table 2). These D-ROIs were areas with significant FA difference between mTBI and control participants (D-ROI1 right anterior corona radiata) or significant association with PCS symptom report (as indexed by RPQ) in participants with mTBI (D-ROI2 splenium and body of corpus callosum). ROI masks were created using the *z*-statistic image (Fig.2B)

In the previous MRS analysis (Dean et al. 2013) there was a significantly reduced Cr/Cho ratio in participants with mTBI compared to controls, and a trend for reduced Cr/Cho in those with PCS. Therefore, the MRS indices used in the correlational analysis consisted of the total Creatine (Cr) concentration and Creatine/Choline (Cr/Cho) ratio within the right dorsolateral prefrontal cortex (DLPFC) for each individual.

## Results

### Demographic and behavioral data

Demographic data are summarized in Table 1. Groups were similar with regard to gender [*χ*^2^ (2, *N* = 25) = 2.3, *P *=* *0.3], age [F(2,22) = 2.6, *P *=* *0.1] and IQ [F(2,22) = 1.4, *P *=* *0.3; mTBI + PCS: 110 ± 2; mTBI-PCS: 119 ± 1; Control: 112 ± 2]. Significant differences were found for handedness [*χ*^2^ (2, *N* = 25) = 8.5, *P *=* *0.02], reflecting more left handers in the control group, and the RPQ [*F*(2,22) = 29.0, *P *<* *0.001], with higher scores in the mTBI + PCS group compared to mTBI-PCS [mean difference (MD) = 14.4, *P *<* *0.001] and control participants [MD = 20.5, *P *<* *0.001].

**Figure 3:**
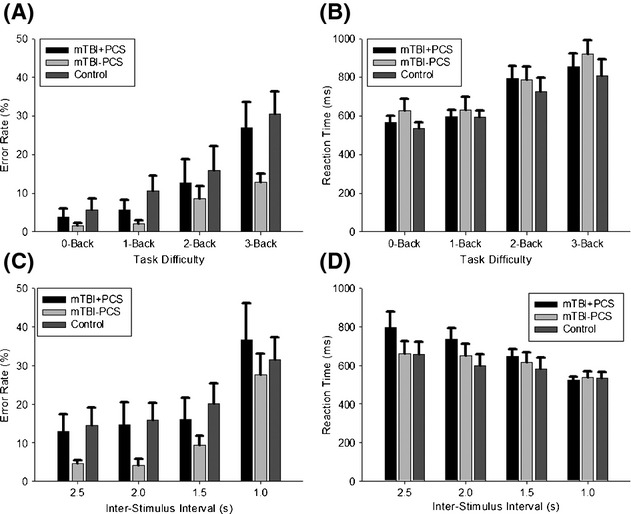
Behavioral data illustrating n-Back error rate (A) and reaction time (B), as well as PVSAT error rate (C) and reaction time (D). Bar graphs represent mean±SEM.

For the behavioral data, there was a main effect of difficulty level for error rate [*n*-Back: *F*(3,58) = 21.8, *P *<* *0.001; PVSAT: *F*(2,45) = 33.4, *P *<* *0.001] and reaction time [*n*-Back: *F*(2,43) = 44.7, *P *<* *0.001; PVSAT: *F*(2,50) = 20.4, *P *<* *0.001], but no difference between the groups (Fig.3) on either measure [error rate: n-Back: *F*(2,21) = 2.4, *P *=* *0.1; PVSAT: *F*(2,22) = 1.3, *P *=* *0.3; reaction time: n-Back: *F*(2,21) = 0.5, *P *=* *0.6; PVSAT: *F*(2,22) = 0.8, *P *=* *0.5] and no group by difficulty interaction (all *P *≥* *0.1).

### Functional imaging data

**Figure 4:**
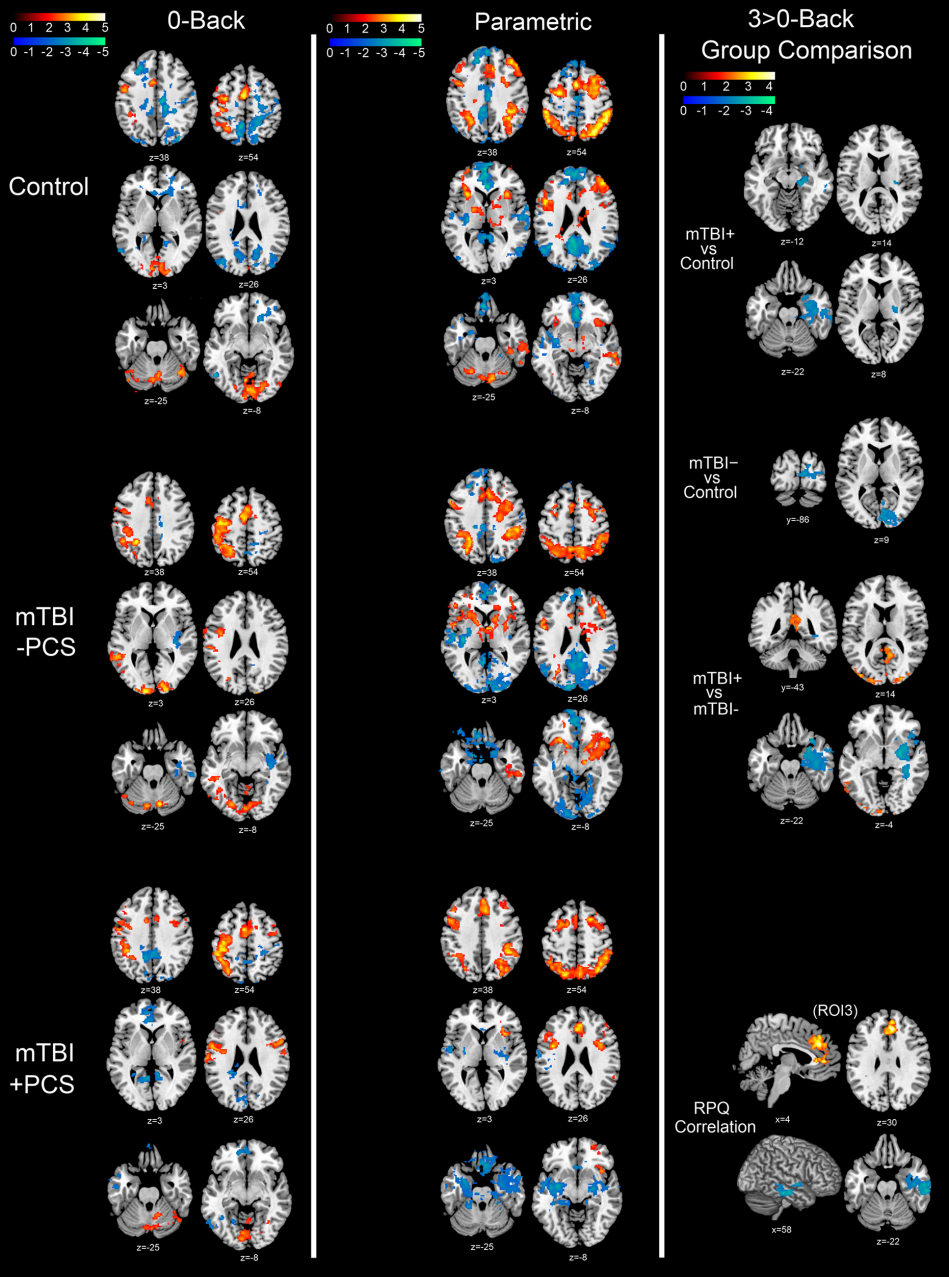
n-Back fMRI contrast maps. Column 1: Main effect of 0-Back block compared to Rest block for Control (top row), mTBI-PCS (middle row) and mTBI + PCS (bottom row). Column 2: Parametric (linearly modeled) increase in BOLD response from least taxing (0-Back) to most taxing (3-Back) for Control (top row), mTBIPCS (middle row) and mTBI + PCS (bottom row). Column 3: Group comparison for the 3 > 0-Back contrast showing significant BOLD response differences between mTBI + PCS and Control, MTBI-PCS and Control, as well as between mTBI + PCS and mTBI-PCS. In addition, areas where 3 > 0-Back contrast significantly correlates with Postconcussion syndrome symptoms as indexed by the Rivermead Postconcussion Symptoms Questionnaire. Red/Yellow Z scale represents areas with significantly increased BOLD response (*Z* > 1.9); Blue/Green *Z* scale represents areas with significantly reduced BOLD response (*Z* > 1.9). Axial, coronal and sagittal plane coordinates indicated under each image. Neurological Orientation (R=R).

**Figure 5:**
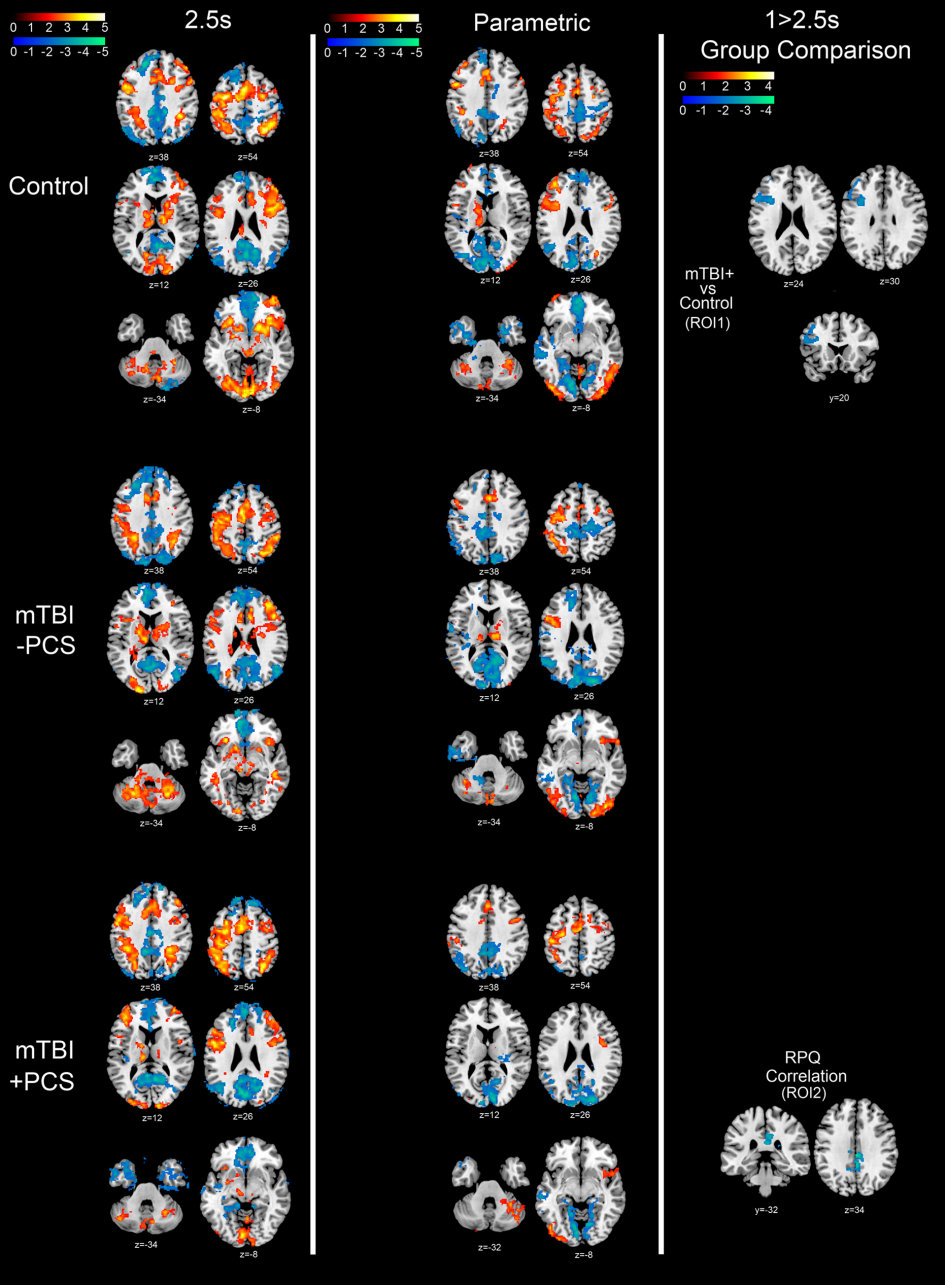
Paced Visual Serial Addition Task fMRI contrast maps. Column 1: Main effect of 2.5s PVSAT block compared to Rest block for Control (top row), mTBI-PCS (middle row) and mTBI + PCS (bottom row). Column 2: Parametric (linearly modeled) increase in BOLD response from least taxing (2.5s PVSAT) to most taxing (1s PVSAT) for Control (top row), mTBI-PCS (middle row) and mTBI + PCS (bottom row). Column 3: Group comparison for the 1 > 2.5s PVSAT contrast showing significant BOLD response differences between mTBI + PCS and Control, MTBI-PCS and Control, as well as between mTBI + PCS and mTBI-PCS. In addition, areas where 1 > 2.5s PVSAT contrast significantly correlates with Postconcussion syndrome symptoms as indexed by the Rivermead Postconcussion Symptoms Questionnaire. Red/Yellow *Z* scale represents areas with significantly increased BOLD response (*Z* > 1.9); Blue/Green *Z* scale represents areas with significantly reduced BOLD response (*Z* > 1.9). Axial, coronal, and sagittal plane coordinates indicated under each image. Neurological Orientation (R=R).

Typical BOLD response patterns were observed for all groups for both 0-Back compared to rest (Fig.4, column 1) and 2.5s PVSAT condition compared to rest (Fig.5, column 1). There was increased BOLD response across groups in motor planning and attention-related areas (e.g., premotor cortex, posterior parietal cortex, supplementary motor area [SMA]), as well as other task-relevant areas (contralateral motor cortex, bilateral cerebellum, bilateral visual cortex). BOLD response also increased during the 2.5s PVSAT task in working memory-related areas (bilateral inferior frontal gyrus (IFG), right DLPFC). Reduced BOLD was observed in areas associated with the default mode network (DMN: posterior cingulate cortex (PCC), precuneus, medial frontal cortex, bilateral parietal regions). As both tasks parametrically increased in difficulty they elicited typical BOLD response increases across groups in the same motor planning, attention, and working memory areas (with additional activity in ventrolateral prefrontal cortex (VLPFC) in the n-Back task), and reduced BOLD response in the areas associated with the DMN (Fig.4, column 2; Fig.5, column 2).

There were no group differences in BOLD response during either the 0-Back or 2.5s PVSAT compared to rest. However, there were significant differences in BOLD response between the groups as task difficulty was increased (3 > 0 Back contrast, Fig.4, column 3; 1 > 2.5s PVSAT, Fig.5, column 3), as well as a significant association between PCS symptom report and BOLD response for these same contrasts. Participants with PCS after mTBI (mTBI + PCS) exhibited a reduced BOLD response in comparison to control participants in motor planning and attention-related areas (contralateral precentral gyrus during PVSAT; Fig.5, Column 3), working memory-related areas (left IFG and left MFG during PVSAT; Fig.5, Column 3) and areas involved in declarative memory and visual processing (medial and inferior temporal lobe during n-Back; Fig.4, Column 3). Furthermore, higher PCS symptom report across participants was associated with a greater BOLD response in attention-related areas (ACC during n-Back) and reduced BOLD response in areas associated with the DMN (PCC and precuneus during PVSAT) and declarative memory, memory encoding, and visual processing (right medial and inferior temporal cortex, parahippocampal area during n-Back). Collectively, these results suggest a reduction of memory-related functional activity in mTBI + PCS participants, and also indicate that there may be some underlying attentional problems.

Other group differences were observed in the n-Back task (Fig.4, Column 2), with mTBI-PCS participants displaying reduced BOLD response compared to controls in visual processing areas (primary/secondary visual cortex and right cuneus) and reduced BOLD response compared to mTBI + PCS participants in areas associated with the DMN (PCC) and visual processing (V1/V2). In addition, mTBI-PCS had a greater BOLD response compared to mTBI + PCS participants in an area associated with declarative memory (medial temporal cortex).

As described in the methods, the activation map around the left IFG and MFG (working memory-related area, ROI 1) and PCC and precuneus (DMN-related area, ROI 2) from the PVSAT contrasts, and the ACC and SMA (attention-related area, ROI 3) in the n-Back contrasts were used as the fMRI Regions of Interest (F-ROI, see Fig.2A) for the correlation analysis in the next section.

### Correlation between functional imaging, structural and metabolic data

The indices from each modality analysis are shown in Table 3.

**Table 3:** Magnetic resonance imaging variables

Group	Age	Gender	fMRI: F-ROI 1	fMRI: F-ROI 2	fMRI: F-ROI 3	DTI: D-ROI 1	DTI:D-ROI 2	MRS:Cr	MRS:Cr/Cho
mTBI + PCS	19	M	16.06	−31.80	36.08	0.22	0.32	6.45	3.28
	19	M	−67.56	−14.36	119.97	0.20	0.31	4.73	3.70
	21	F	−4.92	−28.34	–	0.24	0.39	5.84	3.86
	23	F	−2.16	−40.59	42.90	0.21	0.31	6.20	3.78
	24	F	−6.40	−57.11	70.67	0.26	0.09	5.98	3.37
	26	F	−25.83	−33.00	77.66	0.21	0.26	6.12	4.20
	36	F	−11.56	−20.67	43.94	0.29	0.16	4.99	3.94
	37	F	−4.02	−13.47	50.86	0.28	0.31	6.28	4.38
mTBI-PCS	22	M	20.30	−20.99	7.67	0.23	0.46	5.82	3.63
	23	F	59.45	−28.74	12.77	0.27	0.38	6.48	3.30
	25	F	21.43	−56.04	47.66	0.17	0.40	6.22	3.47
	26	F	32.97	−24.76	54.67	0.27	0.36	6.17	3.88
	29	M	44.17	−25.76	41.60	0.17	0.46	6.41	3.97
	29	M	21.30	10.55	−6.11	0.28	0.64	4.52	3.93
	33	M	−5.02	−11.61	3.27	0.22	0.32	6.05	3.54
	39	M	27.86	16.44	−40.02	0.40	0.48	6.33	3.52
Control-PCS	18	F	34.98	29.01	−3.49	0.25	0.39	6.31	3.65
	18	M	64.26	−12.78	27.74	0.32	0.34	5.83	3.84
	19	M	67.82	−28.68	20.00	0.23	0.22	6.37	3.66
	20	F	31.73	−8.89	−10.23	0.30	0.34	5.76	3.54
	20	M	99.56	2.10	−2.50	0.21	0.50	6.53	3.87
	22	M	45.27	−6.05	−35.03	0.34	0.54	6.70	4.11
	23	F	34.54	16.72	27.71	0.27	0.51	6.85	4.24
	25	F	34.82	−19.52	23.80	0.38	0.54	5.99	4.55
	32	F	18.76	67.59	−4.48	0.44	0.35	6.05	3.64

Values used in correlation analysis. fMRI *z*-statistic from Regions of Interest (F-ROI, see Fig.2A), DTI Fractional Anisotropy from Regions of Interest (D-ROI, See Fig.2B) and MRS Concentrations from right Dorsolateral Prefrontal Cortex.

**Figure 6:**
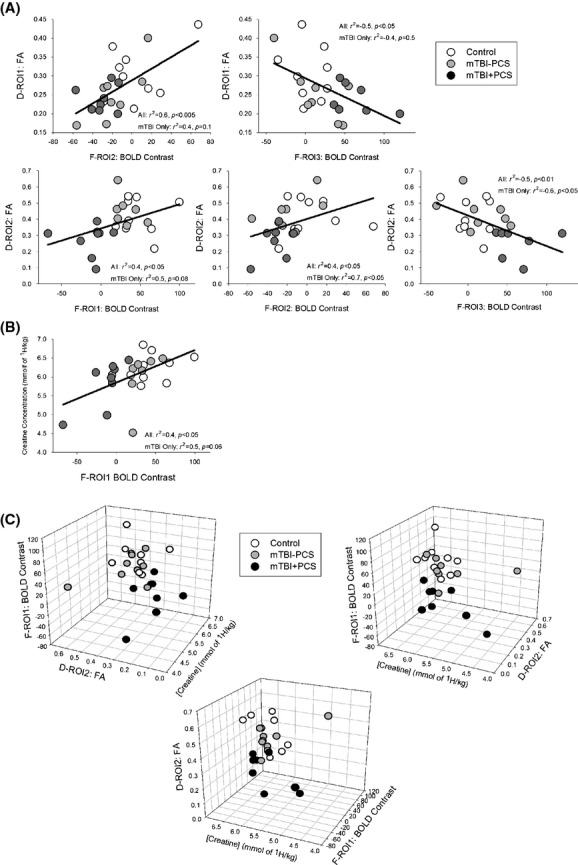
Correlations between functional data and (A) structural data, (B) metabolic data or (C) both structural and metabolic data in the same participants. The 3D scatterplot (C) illustrates the relationship between FROI1 (Left IFG/MFG BOLD Contrast), D-ROI2 (Splenium of Corpus Callosum FA) and Creatine Concentration (in rDLPFC) from three different perspectives.

A reduced BOLD response in working memory areas (left IFG/MFG: F-ROI 1 during PVSAT 1 > 2.5s) was associated with lower fractional anisotropy (FA) in the splenium of the corpus callosum (D-ROI 2, Fig.6A, *r*(21) = 0.4, *P *=* *0.039) and reduced creatine concentration in right DLPFC (Fig.6B, *r*(21) = 0.5, *P *=* *0.013) across participants. These correlations were not significant in mTBI participants alone (D-ROI 2: *r*(12) = 0.5, *P *=* *0.075; Creatine: *r*(12) = 0.5, *P *=* *0.06).

Lower FA in the splenium of the corpus callosum (D-ROI 2) was also associated with a reduced BOLD response in DMN-related areas (PCC and precuneus, F-ROI 2 during PVSAT 1 > 2.5s: *r*(21) = 0.4, *P *=* *0.042) and increased BOLD response in attention-related areas (ACC and SMA, F-ROI 3 during n-Back 3 > 0-Back: *r*(21) = −0.5, *P *=* *0.007) both across participants, and for mTBI participants only (F-ROI 2: *r*(21) = 0.7, *P *=* *0.012; F-ROI 3: *r*(12) = −0.6, *P *=* *0.017).

Additional function–structure correlations (Fig.6A) were seen between lower FA in right anterior corona radiata (D-ROI 1, Fig.2B) and reduced BOLD response in F-ROI 2 (*r*(21) = 0.4, *P *=* *0.003) as well as increased BOLD response in F-ROI 3 (*r*(21) = −0.5, *P *=* *0.012). These comparisons were not seen for mTBI participants alone (F-ROI 2: *r*(12) = 0.4, *P *=* *0.12; F-ROI 3: *r*(12) = −0.4, *P *=* *0.17).

There was no association between FA in D-ROI 1 and BOLD response in F-ROI 1 (*r*(21) = 0.3, *P *=* *0.18). Creatine concentration in DLPFC did not correlate with BOLD response change in F-ROI 2 (*r*(21) = 0.01, *P *=* *0.95) or F-ROI 3 (*r*(21) = −0.3, *P *=* *0.14).

## Discussion

There have been relatively few studies using fMRI to investigate mTBI, with the majority investigating the subacute or acute phase post-injury (McDonald et al. 2012). The present data expand the existing literature by demonstrating functional changes during performance of two cognitive tasks in the long term (>1 year) after injury, and demonstrate for the first time that these functional changes correlate with data from other imaging modalities as well as self-reported PCS symptoms. As hypothesized, individuals with mTBI and ongoing PCS exhibited the greatest differences compared to controls, with reduced BOLD response in working memory-related areas (left IFG/MFG during PVSAT) and declarative memory and visual processing areas (right inferior/medial temporal areas during n-Back). Furthermore, increased PCS symptom report correlated with reduced BOLD response in declarative memory and visual processing areas (right inferior/medial temporal areas during n-Back), DMN-related areas (PCC and precuneus during PVSAT), as well as increased BOLD response in attention-related areas (ACC during n-Back).

**Figure 7:**
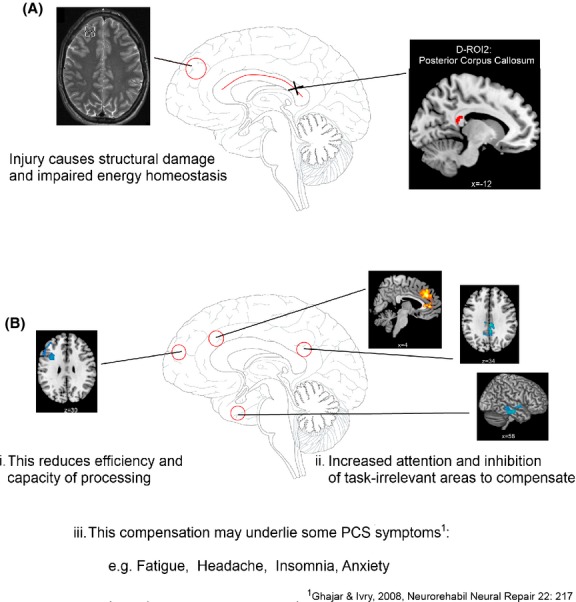
A model illustrating how the structural and metabolic changes after injury (A) may cause the functional changes and compensation mechanisms during high cognitive load (B) which in turn may underlie some of the ongoing PCS symptoms in those participants with mTBI and ongoing PCS.

These functional changes correlated with structural integrity (DTI) and alterations in metabolism (MRS) such that a smaller BOLD response increase in left IFG/MFG for the hardest task was associated with lower FA in the splenium of the corpus callosum as well as lower creatine concentration in rDLPFC. Reduced FA in the splenium of the corpus callosum was also seen in those with reduced BOLD response in PCC and precuneus, and increased BOLD response in ACC. Our data suggest that mTBI participants with persistent PCS are compensating for reduced capacity caused by structural and metabolic changes after injury by top-down regulation of attention and deactivation of task-irrelevant areas during difficult tasks (see Fig.7). These results and conclusions will be discussed in detail below.

### fMRI data: n-Back

A number of studies have used n-Back to investigate the acute phase after injury, and have found smaller increases in BOLD response compared to controls when performing the most difficult task. These functional changes are typically found in more prefrontal neural areas, even in the absence of behavioral differences (McAllister et al. 1999, 2001; Chen et al. 2012). Evidence for functional changes in the chronic stage after injury is limited, with only one published study in this area. This study reported no functional or behavioral differences (Elbin et al. 2012).

In the present study there were no behavioral differences in the n-Back data, but some significant group differences in BOLD response modulation during the hardest task (3-Back) compared to the easiest (0-Back). The modulation of BOLD response in right temporal areas (inferior and medial temporal) was lower in mTBI + PCS participants compared to both mTBI-PCS and control participants. In addition, this attenuated BOLD response correlated with PCS symptom report across participants. A previous study found reduced task-related deactivation of medial temporal lobe (MTL) during the n-Back correlated with increased severity of injury in mTBI in the acute stage (Stulemeijer et al. 2010). It was suggested that mTBI may interfere with the disengagement typically exercised by the MTL during the n-Back. However, there was greater task-related deactivation in those with greater symptom report in our chronic mTBI sample.

The explanation for the discrepancy between our results and previous research could be linked with the association between increased BOLD response in ACC and greater PCS symptom report. This suggests that participants with ongoing PCS may require higher levels of attention and monitoring, as well as greater top-down suppression of task-irrelevant information, to achieve the same task performance. As this study was conducted on chronic (>1 year) mTBI participants, it could be that those participants with ongoing PCS have developed mechanisms to cope with their deficit by increasing attention so as to increase task-related deactivation of right MTL.

### fMRI data: PVSAT

There are no studies which have used fMRI data acquired during PVSAT performance to investigate mTBI. This is surprising, as the paced auditory serial addition task (PASAT) was originally designed to investigate cognitive difficulties after TBI and is sensitive to changes after mTBI (Fos et al. 2000). Moreover, the PVSAT is suitable for fMRI, and has been in existence for a number of years (Lazeron et al. 2003). However, two studies have explored fMRI alterations after severe diffuse axonal injury (Maruishi et al. 2007) and moderate to severe TBI (Christodoulou et al. 2001) in the postacute stage. There was only one experimental condition used for both studies (2s interstimuli interval), with one study using visual stimuli [PVSAT; (Maruishi et al. 2007)], whilst the other used auditory stimuli [PASAT; (Christodoulou et al. 2001)]. The present study therefore represents the first experiment examining fMRI activity in mTBI participants performing a parametric PVSAT task with four difficulty levels (2.5s, 2s, 1.5s, 1s).

The two previous studies observed greater BOLD response in the TBI group, particularly in the right hemisphere (Christodoulou et al. 2001), and right IFG/MFG (Maruishi et al. 2007). Conversely, the present study observed a smaller BOLD response increase in left IFG, left MFG and precentral gyrus during the hardest task for mTBI participants with ongoing PCS compared to controls. Whereas the control participants utilize the left prefrontal cortex to a greater degree in the harder task, those with mTBI and ongoing PCS have no extra capacity. mTBI + PCS participants may have reached the limits of their working memory capacity at the easiest PVSAT condition, with left prefrontal activity observed in the 2.5s condition, but no significant increase with difficulty level (see Fig.5, column 1 and 2). These data are contrary to the previous studies, but they examined more severely injured participants [with mean Glasgow Coma Scale of 5.4 (Maruishi et al. 2007) and 5.7 (Christodoulou et al. 2001)], and only one PVSAT condition (2s), so may not be directly comparable. More subtle differences are expected in such a mildly injured group in the long term after injury in comparison to more severe TBI.

In addition to differences in prefrontal BOLD response, this study found that participants with higher PCS symptom scores exhibit a greater reduction in BOLD response in DMN-related areas (PCC, precuneus) and the right thalamus when performing the hardest compared to the easiest PVSAT condition. Conflicting results have been obtained by previous studies on DMN alterations after mTBI, with reduced DMN connectivity seen at rest (Mayer et al. 2011; Johnson et al. 2012; Zhang et al. 2012), but both lower (Bonnelle et al. 2012; Mayer et al. 2012) and higher task-induced DMN deactivation (Sharp et al. 2011). However, only the study which found greater task-induced deactivation investigated performance in chronic TBI (Sharp et al. 2011). Furthermore, a negative association between PCS symptom report and lower BOLD response in DMN-related areas has been found previously in participants performing the n-Back task (Pardini et al. 2010). These previous studies are both consistent with our current findings, which in turn lend support to the theory that participants with mTBI and ongoing PCS need to attend to the task more to achieve the same performance and so exhibit greater deactivation of task-irrelevant areas. Indeed, previous research has shown that increased DMN activation during tasks (particularly in the PCC and precuneus) is associated with sustained attention impairments after TBI (Bonnelle et al. 2011). A greater reduction of BOLD response in DMN areas in those with greater PCS therefore suggests a greater degree of top-down attention is utilized in these participants as a coping strategy in the long term after injury.

### fMRI data summary

Summarizing across tasks, the data presented here suggest that participants with mTBI and ongoing PCS have limited working memory capacity (lower BOLD increase in left IFG/MFG), and compensate for this by greater attention and performance monitoring (greater BOLD increase in ACC) and reduction of activity in task-irrelevant areas (medial temporal lobe and DMN). These functional changes are observed even in the absence of behavioral differences (Fig.3), as has been seen in previous studies (McAllister et al. 2001; Stulemeijer et al. 2010; Witt et al. 2010; Chen et al. 2012) indicating that fMRI may be more sensitive to subtle changes after mTBI.

It has previously been suggested that a large scale disorder of attention underlies the symptoms seen after TBI (Ghajar and Ivry 2008), with enhanced top-down control of attention necessary to compensate for microstructural damage causing variability in white matter transmission speed. In this model, the compensation is through greater prefrontal activation, whereas in our study we see greater ACC activation and a relative reduction in prefrontal areas. This difference may be due to the recruitment of mild TBI participants, but also that participants had practised these tasks before in a previous study (Dean and Sterr 2013). Prefrontal areas are thought to be hyperactive after mTBI to aid in practise of a task (Hillary et al. 2010, 2011; Medaglia et al. 2012), and reduce after the task is learned (Medaglia et al. 2012). The performance of mTBI participants is similar to controls in these tasks, and this study may only demonstrate the compensatory functional changes required to perform the task at this level and not the initial prefrontal hyperactivation when practising a novel taxing task.

### Multimodal data

The model of large scale disorder of attention after TBI (Ghajar and Ivry 2008) posits that primary symptoms (decreased attention and memory) are related to a predictive timing deficit caused by microstructural axonal damage and secondary symptoms (such as fatigue and headaches) are a result of functional compensation for these deficits. Increased variability in white matter transmission speed causes variation in behavioral performance, and is compensated for by enhancing top-down control of attention.

We found evidence for such a relationship in this study, with reduced structural integrity in right anterior corona radiata (D-ROI 1) and the splenium of the corpus callosum (D-ROI 2) associated with enhanced activation of attention-related areas (F-ROI 3, ACC) and deactivation of task-irrelevant areas (F-ROI 2, PCC and precuneus). In this way, structural damage is associated with greater top-down control of attention during cognitive tasks. Furthermore, reduced BOLD response in prefrontal working memory areas (left IFG/MFG) was associated with reduced structural integrity in the splenium of the corpus callosum and altered energy homeostasis (reduced creatine concentration in rDLPFC). This suggests a possible mechanism for the limited working memory capacity observed in participants with mTBI. In summary, the neuroimaging indices presented here seem to fit with the model of a large-scale disorder of attention after TBI (Ghajar and Ivry 2008), to offer a possible mechanism of how the structural and metabolic damage after injury may cause the functional changes which in turn may underlie the ongoing PCS symptoms observed (see Fig.7).

Previous studies combining fMRI and DTI have revealed a correlation between structural integrity (apparent diffusion coefficient, ADC) and BOLD response in bilateral DLPFC (Zhang et al. 2010), and a correlation between symptom report (major depression) and structural integrity (FA) in long white matter tracts (Matthews et al. 2010). There have been many studies reporting microstructural damage to long white matter tracts (Rutgers et al. 2008; Smits et al. 2011; Kasahara et al. 2012; Ling et al. 2012), and metabolic changes (see (Lin et al. 2012) for a review) after mTBI. However, this is the only study to have combined fMRI, DTI, and MRS and found interactions between these three indices which enable a working theory of the underlying causes of persistent PCS.

In addition to investigating the mechanisms of injury, multimodal imaging may be more sensitive in the detection of the subtle deficits likely to be observed after mTBI. Furthermore, it may help counter the inherent variability of injury in mTBI by allowing individualized profiling across a set of parameters, and thus individual prognosis (Gonzalez and Walker 2011; Hunter et al. 2012; Irimia et al. 2012; McDonald et al. 2012; Shenton et al. 2012; Slobounov et al. 2012). As a result of the mechanism of injury, some individuals may demonstrate a greater difference in one specific modality (e.g., DTI), whilst another may exhibit more of a difference in another modality (e.g., MRS). This would mean that by looking at one modality and averaging across participants, you may be missing subtle changes due to this heterogeneity (Rosenbaum and Lipton 2012). An example 3D plot of the fMRI data from F-ROI1, the DTI data from D-ROI 2, and the creatine concentration from rDLPFC is illustrated in Figure6C. The data are correlated with each other, so there are no subtle changes being masked by heterogeneity in the modalities in this example. However, there is a much clearer delineation between the three groups using the three data sources in 3D space, which may result in much more accurate in group categorization (diagnosis and prognosis) using multidimensional discriminant analysis (Sato et al. 2009; Oliveira et al. 2010). This style of analysis may also help find subtle changes where conventional analyses are not successful.

## Conclusions

This study presents novel research which reveals participants in the long term after injury with persistent PCS exhibit increased top-down attentional regulation of task-irrelevant areas and reduced working memory capacity. Furthermore, the combined use of functional, structural, and metabolic data in the same sample helped to conclude that these functional changes may be compensating for underlying structural and metabolic alterations which have increased the variability of white matter transmission and reduced the capacity of those participants with persistent PCS to cope with increasing cognitive load. This compensation may also contribute to secondary PCS symptoms such as fatigue and headaches. In addition, the use of more sensitive neuroimaging tools such as fMRI, DTI, and MRS can help improve detection of brain abnormalities after mTBI, but when used in combination they may offer even greater improvements in diagnosis and prognosis.
